# The PERCIVAL detector: first user experiments

**DOI:** 10.1107/S1600577522010347

**Published:** 2023-01-01

**Authors:** J. Correa, M. Mehrjoo, R. Battistelli, F. Lehmkühler, A. Marras, C. B. Wunderer, T. Hirono, V. Felk, F. Krivan, S. Lange, I. Shevyakov, V. Vardanyan, M. Zimmer, M. Hoesch, K. Bagschik, N. Guerrini, B. Marsh, I. Sedgwick, G. Cautero, L. Stebel, D. Giuressi, R. H. Menk, A. Greer, T. Nicholls, W. Nichols, U. Pedersen, P. Shikhaliev, N. Tartoni, H. J. Hyun, S. H. Kim, S. Y. Park, K. S. Kim, F. Orsini, F. J. Iguaz, F. Büttner, B. Pfau, E. Plönjes, K. Kharitonov, M. Ruiz-Lopez, R. Pan, S. Gang, B. Keitel, H. Graafsma

**Affiliations:** a Deutsches Elektronen-Synchrotron DESY, Notkestrasse 85, 22607 Hamburg, Germany; b Center for Free-Electron Laser Science CFEL, Deutsches Elektronen-Synchrotron DESY, Notkestrasse 85, 22607 Hamburg, Germany; c Helmholtz Zentrum Berlin HZB, Hahn-Meitner-Platz 1, Berlin, Germany; d The Hamburg Centre for Ultrafast Imaging CUI, Luruper Chaussee 149, 22761 Hamburg, Germany; e Science and Technology Faculties STFC, Rutherford Appleton Laboratory RAL, Didcot, United Kingdom; f Elettra Sincrotrone Trieste, Trieste, Italy; g University of Saskatchewan, Saskatoon, Saskatchewan, Canada S7N 5A2; h Observatory Sciences Ltd, Cambridge, United Kingdom; i Diamond Light Source, Didcot, United Kingdom; j Pohang Accelerator Laboratory PAL, Pohang, Gyeongbuk 37673, Republic of Korea; k Synchrotron SOLEIL, Saint Aubin, France; l Max-Born-Institute MBI, Max-Born-Straße 2A, Berlin, Germany; m Mid Sweden University, Sundsvall, Sweden; University of Tokyo, Japan

**Keywords:** X-ray detectors, soft X-rays, ptychography, holographic imaging, XPCS, detectors

## Abstract

The PERCIVAL detector is a CMOS imager designed for the soft X-ray regime at photon sources. Although still in its final development phase, it has recently seen its first user experiments: ptychography at a free-electron laser, holographic imaging at a storage ring and preliminary tests on X-ray photon correlation spectroscopy. The detector performed remarkably well in terms of spatial resolution achievable in the sample plane, owing to its small pixel size, large active area and very large dynamic range; but also in terms of its frame rate, which is significantly faster than traditional CCDs. In particular, it is the combination of these features which makes PERCIVAL an attractive option for soft X-ray science.

## Abstract 2

PERCIVAL is a detector system specifically designed for the soft X-ray regime. Although still in a development phase, it has already served its first user experiments at both a storage ring and also a free-electron laser. The device performed remarkably well in all the different techniques tested: ptychography, holography and also X-ray photon correlation spectroscopy. The results of these tests are presented.

## 1. Introduction

The Pixelated Energy Resolved CMOS Imager, Versatile And Large (PERCIVAL) detector is an imager specifically designed for the soft X-ray regime. With its 2 Megapixels, 27 µm pixel size and 4 cm × 4 cm active area (extendable to 8 Megapixels and clover-leaf configurations), PERCIVAL can provide images with high spatial resolution. Moreover, its fast readout is designed to reach speeds of up to 300 frames per second. Applying regions of interest (ROIs) within the active area could allow the frame rate of the system to reach the kilohertz regime. Once fully optimized, its adaptive gain capability will provide a dynamic range spanning 15 e^−^ to 3.5 Me^−^, or several tens of thousands of photons at *E*_ph_ = 250 eV per pixel per frame. The development, jointly carried out by six research centers [DESY, Rutherford Appleton Laboratory (RAL/STFC), DLS, Elettra, PAL and SOLEIL], aims to increase the scientific yield of today’s free-electron lasers (FELs) and storage ring (SR) sources in the soft X-ray regime.

The main objectives of the PERCIVAL project have already been defined elsewhere (Wunderer *et al.*, 2014 ▸). The whole development process, from small prototypes to the full system and its characterization, have also been published over the years (Correa *et al.*, 2016*a* ▸,*b* ▸). Moreover, the status, capabilities and drawbacks of the current system have been previously reported (Wunderer *et al.*, 2019 ▸; Marras *et al.*, 2021 ▸). In a nutshell, and as can be seen in Table 1 ▸, the current system is limited in terms of frame rate to 83.3 Hz instead of the envisaged 300 Hz, which is primarily because crosstalk in the present, first version of the sensor forced less-aggressive parallelization of digitization and streamout on-chip, and the presence of some non-linearities in the output data. Fine tuning of the system has allowed us to partially overcome some of the issues mentioned until a newer version of the sensor is available. This is partially done by the use of fixed gain modes with limited dynamic range and better noise performance.

In that context, and in order to explore its potential even at this stage of development, several experiments have been performed recently. First, in collaboration with users groups from Helmholz Zentrum Berlin (HZB) and Max–Born Institute (MBI), holographic imaging of the magnetic texture in a chiral magnetic material was performed at the P04 XUV beamline at PETRA III. A full account of the experiment and some of the preliminary results can be found in Section 2. Moreover, ptycho-imaging was performed at the FL24 beamline at the FLASH2 facility. The experiment is described and results are given in Section 3.

Both experiments benefited from the combination of very large dynamic range and high frame rate supplied by the current version of the system. In this paper, we show the capabilities of the PERCIVAL detector at the beamlines and show in which ways the aforementioned as well as other techniques in the soft X-ray regime could benefit from this development. In particular, a brief introduction to X-ray photon correlation spectroscopy (XPCS) and the advantages it could achieve using the PERCIVAL detector are given in Section 4.

## 2. Fourier transform holography and phase retrieval of magnetic materials

X-ray Fourier transform holography (FTH) is a coherent diffractive microscopy technique that allows retrieval of the complex sample transmission function of a thin sample from a coherent scattering pattern without using complex iterative algorithms (McNulty *et al.*, 1992 ▸; Eisebitt *et al.*, 2004 ▸). FTH is a full-field technique and is inherently drift-free, making it highly stable against setup vibrations and therefore ideal for dichroic imaging where multiple images with different X-ray polarisations need to be acquired with a constant field of view. This property makes it highly suitable for imaging with magnetic contrast, exploiting the X-ray magnetic circular dichroism (XMCD) (Stöhr *et al.*, 2006 ▸). FTH is an efficient and highly stable experimental technique for magnetic microscopy and, for instance, has enabled recent advances in the field of magnetic skyrmions (Turnbull *et al.*, 2021 ▸; Caretta *et al.*, 2018 ▸; Gerlinger *et al.*, 2021 ▸) and magnetic multilayers (Büttner *et al.*, 2017 ▸).

Similar to ptychography, the quality of FTH results heavily depends on the detector performance. Compared with CCDs still used as standard area detectors for soft X-ray imaging, PERCIVAL provides an improvement of the spatial resolution of FTH images via its significantly larger dynamic range, enabling efficient recording of low scattering intensity signal at high diffraction angles without overexposing the detector at low scattering angles. Moreover, the fast camera readout will reduce detector dead-times during imaging experiments and therefore significantly increase their productivity.

The FTH experiment using soft X-ray detection with PERCIVAL was carried out at the PETRA III synchrotron radiation source in Hamburg, at the P04 beamline (Viefhaus *et al.*, 2013 ▸). As a prototypical sample, we used a Pt(3)/[Pt(2 nm)/Co(0.8 nm)/Cu(0.5 nm)]_15_/Pt(2) magnetic multilayer grown on a 200 nm-thick, X-ray-transparent silicon-nitride membrane. The magnetic film exhibits perpendicular magnetic anisotropy, resulting in the formation of nanometre-scale magnetic domains with ‘up’ or ‘down’ out-of-plane magnetization after demagnetization by magnetic field cycling.

On the opposite side of the substrate, we produced a standard holography mask in a 700 nm-thick, X-ray-opaque Au/Cr multilayer. The mask comprises a 1 µm large circular aperture defining the field of view and four reference holes with varying diameters (49 to 190 nm); the latter provides the source for the reference wave.

**Figure 1:**
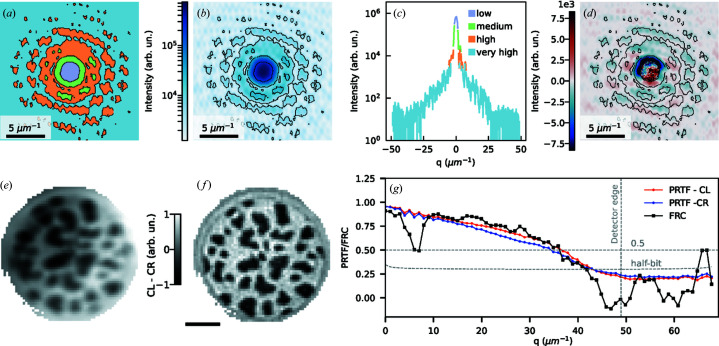
Results of FTH and phase retrieval with the PERCIVAL detector. (*a*) Map of fixed gains used for the composed final hologram (central part only). For color definition see the legend in (*c*). (*b*) Hologram [same crop as in (*a*)] assembled from all data recorded with different gain settings and left-handed polarised soft X-rays. Contour lines mark transitions from one fixed gain region to the next. (*c*) Line scan of the hologram in (*b*) through its center. Pixel values using different gains are depicted using different colors. (*d*) Difference hologram corresponding to (*b*). (*e*) FTH reconstruction of (*d*) revealing magnetic domains inside the field of view and (*f*) corresponding phase-retrieved reconstruction. Scale bar is 300 nm. (*g*) Phase-retrieval transfer function and Fourier ring correlation of the reconstruction in (*f*). Gray dashed lines indicate the 0.5 and half-bit thresholds.

In the experiment, the PERCIVAL detector, placed 33 cm from the sample, recorded coherent scattering patterns with circularity left- and right-handed synchrotron radiation tuned to the Co *L*_3_-edge (λ = 1.59 nm). Due to the restricted capabilities of the then-current version of the PERCIVAL detector, only fixed gain operation was available at the time of the experiment (July 2020). We acquired four sets of data for both polarisations, each using a different fixed gain to address regions of the scattering patterns with different intensities. Every set contained 100 frames with an exposure time of 180 ms each, which we summed into a single image before proceeding. We then merged images acquired with different gains into a single hologram by pixel-wise selection of the recording with the highest gain available for which the pixel was not saturated. The process is illustrated in Fig. 1 ▸(*a*), showing a map of the gain used for every pixel. At high scattering angles, the small signal was amplified with the highest gain while lower and lower gains are used towards the center of the scattering patterns to accommodate for the more intense signal and avoid saturation. This results in a composite hologram with significantly increased dynamic range (up to six orders of magnitude) and signal-to-noise ratio, as shown in Figs. 1 ▸(*b*) and 1 ▸(*c*). Future versions of the PERCIVAL detector will offer the capability to record holograms over the full dynamic range in a single acquisition based on adaptive gain switching.

To isolate magnetic information, we subtracted two high-dynamic-range holograms taken with opposite circular polarisation and obtained the difference hologram presented in Fig. 1 ▸(*d*). Although the high dynamic range of the PERCIVAL detector allowed us to record the center of the scattering pattern without covering it with a beamstop, it is apparent that the pixels with the lowest gain, located at said center, present a much lower signal-to-noise ratio compared with pixels with higher gain. An inverse Fourier transform of the difference hologram results in a holographic image reconstruction as presented in Fig. 1 ▸(*e*). The image reveals a nanometre-scale pattern of opposed magnetized domains which is typical for this kind of ferromagnetic multilayers. Although the holographic reconstruction method provides a direct and immediate solution to the phase problem, the spatial resolution remains limited by the size of the reference holes in the holographic mask (Pfau & Eisebitt, 2016 ▸). To further improve the image resolution and contrast, we employed phase-retrieval algorithms for coherent diffractive imaging. The methods iteratively recover the phase information from the scattering pattern using some *a priori* information about the sample, such as its physical shape and extent. In our case, we derived the shape and size of the circular field of view and the four reference holes by thresholding the FTH reconstruction, and adopted a protocol suitable for dichroic imaging (Kfir *et al.*, 2017 ▸). First, we retrieved the phase from the left-polarised scattering pattern using a combination of the relaxed-averaged alternating reflection (RAAR) and the solvent flipping (SF) algorithms (Marchesini, 2007 ▸), using 700 and 50 iterations, respectively. Then, the resulting left-polarised phase was overlayed with the right-polarised scattering pattern and the right-polarised phase was retrieved with 50 iterations of the SF algorithm, ensuring alignment between the two different polarisation images. The result is shown in Fig. 1 ▸(*f*). The domains are sharper and more defined than in the FTH reconstruction, providing an accurate map of the sample magnetization. We evaluated the resolution of our reconstruction using the phase-retrieval transfer function (Chapman *et al.*, 2006 ▸) and the Fourier ring correlation (Saxton & Baumeister, 1982 ▸) method, and the results are displayed in Fig. 1 ▸(*h*). The resolution was evaluated using the 0.5 and half-bit thresholds, respectively, yielding maximum recorded spatial frequencies of 36 µm^−1^ and 42 µm^−1^, corresponding to resolutions of approximately 27 nm and 24 nm, respectively.

In summary, we used the PERCIVAL detector to acquire FTH images of a magnetic multilayer, exploiting the different gain settings of the detector to record the entire scattering pattern without using beamstops to protect the camera from the direct beam. Moreover, we successfully applied phase-retrieval algorithms to the recorded data to increase the resolution to values unattainable using FTH alone. We conclude that the PERCIVAL detector is suitable for resonant coherent imaging with both FTH and holographically aided iterative phase retrieval.

## 3. Ptychography at an FEL

Microscopic imaging in the soft X-ray regime enables the study of complex material properties in a wide range of fields and techniques. Among the microscopic methods, ptychography X-ray imaging combines the advantages of scanning transmission X-ray microscopy (STXM) with the higher contrast and spatial resolution of recently developed techniques of coherent diffraction imaging (CDI). Ptychography is a scanning diffraction imaging technique that can simultaneously retrieve the sample transmission function and an unknown illuminating probe (Pfeiffer, 2018 ▸; Thibault *et al.*, 2008 ▸). Soft X-ray ptychography in particular is able to reveal local chemical environment changes, magnetic states and bond orientation (Sun *et al.*, 2021 ▸; Shi *et al.*, 2016 ▸; Gao *et al.*, 2020 ▸). The emergence of FELs, offering highly intense and ultra-short coherent laser-like pulses, has allowed diffraction imaging to flourish and achieve high-resolution images of a large variety of samples.

Utilizing pulses of an FEL for ptychography potentially demands a twofold process covering data acquisition and data analysis. FELs, based on self-amplified spontaneous emission, are of stochastic nature and generate pulses with spatial shot-to-shot fluctuations on the order of micrometres. For a precise data analysis and structural interpretation, the shot-to-shot fluctuations should be computationally integrated into the retrieval process. Recently, advances in computational science have linked the concept of automatic differentiation (AD) into ptychography imaging (Kandel *et al.*, 2019 ▸). The AD technique permits the integration and optimization of fluctuating parameters, such as transverse coherence and position instability, into the retrieval algorithm. Recently the use of AD ptychography for imaging and shot-to-shot ptychographical beam characterization at the EUV SASE FEL was demostrated for the first time by Kharitonov *et al.* (2021 ▸).

The data acquisition strongly relies on, among other factors, the capabilities of the detector. A large field of view, given by a large chip size combined with small pixel size to adequately sample diffraction patterns, can notably improve the retrieval resolution. Also, a high dynamic range of the detector allows the capture of weak high-angle-scattered photons without needing to block the strong un-scattered beam. This simultaneously allows us to reliably measure the lower- and higher-frequency components of the sample, thus improving the convergence and overall resolution of the reconstruction (Rose *et al.*, 2017 ▸). A high-frame-rate data acquisition can speed up the raster scanning process, and better utilize light pulses of the high-repetition-rate FELs. Additionally, it minimizes the time required for the full ptychographical measurement, making it easier to preserve sensitive samples during the measurement. The total X-ray dose absorbed by the sample may cause degradation that can prevent convergence of the ptychography retrieval, as well as mislead the data interpretation. A high quantum efficiency of the detector helps to reduce the total dose and improve the quality of retrievals. The PERCIVAL meets all the above-mentioned requirements for the detector and improves both data quality and acquisition speed for ptychography at FELs.

**Figure 2:**
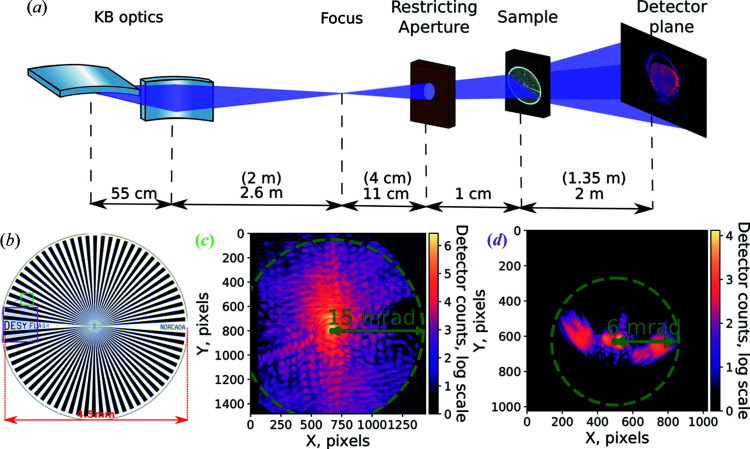
(*a*) Schematic outline of the experimental setup used for the experiment described by Kharitonov *et al.* (2021 ▸). The FEL beam was focused using KB mirrors, thus creating a divergent probe geometry. A restricting aperture was used to limit spatial fluctuations. The sample was placed after the focus. Values in brackets correspond to the setup used with the PERCIVAL detector in 2020. (*b*) Optical microscopy image of the sample. Two scanned areas, one highlighted in a magenta square and one in green, were used. The magenta area indicates non-symmetric structure made up of letters and the green area bright and dark lines that radiate from a common center. Comparison of typical diffraction patterns measured with (*c*) the PERCIVAL detector and (*d*) the CCD camera reported by Kharitonov *et al.* (2021 ▸). Photons with approximately two orders of magnitude intensity and higher diffraction angles were captured with the PERCIVAL detector compared with the typical measurements in (*d*). Achieving the same dynamical range in (*d*) demands an increase in intensity and, consequently, a beamstop. The green scales in (*c*) and (*d*) show the maximum measured angle of diffraction in Fourier (*q*) space.

The first ptychography experiment utilizing the PERCIVAL detector was performed at the FL24 beamline (Plönjes *et al.*, 2016 ▸; Ruiz-Lopez, 2019 ▸; Faatz *et al.*, 2016 ▸) at a wavelength of λ = 13.5 nm corresponding to a photon energy of *E*_ph_ = 91.8 eV. This energy is outside the primary single-photon-counting range of PERCIVAL. However, its thin entrance window allows the user to perform measurements at lower energies as long as single-photon sensitivity is not required. The ptychography dataset measured with PERCIVAL was compared with a similar measurement conducted with an ANDOR iKon-L SO camera used to reproduce the results by Kharitonov *et al.* (2021 ▸). For both measurements, the same test sample was used: a 4.5 mm × 4.5 mm Siemens star pattern fabricated from 190 nm-thick Au and 10 nm-thick Cr layers on a 150 nm-thick SiN membrane. The experimental setup is illustrated in Fig. 2 ▸(*a*) and the Siemens star in Fig. 2 ▸(*b*). The beam was focused using bendable Kirkpatrick–Baez (KB) mirrors. The sample was placed 4 cm behind the focus. A beam-defining aperture was used to restrict possible spatial fluctuations of the probe and was placed 1 cm before the sample. A measurement of 11 × 11 scan positions was performed along the sample for the reconstruction. In total, 10 images were measured per position and the brightest one was selected as the representative for the particular position to minimize the influence of the FLASH fluctuations. The measurements were performed in single-bunch mode using the fast shutter to select individual light pulses from the FLASH pulse train. The images were taken at a rate of 10 frames per second using PERCIVAL (limited by the FLASH base rate), and 0.9 frames per second using the CCD camera, limited by the camera itself. Thus, the measurement times without the motor movement (4 min) were 2 min and 22 min for the PERCIVAL detector and the CCD camera, respectively.

**Figure 3:**
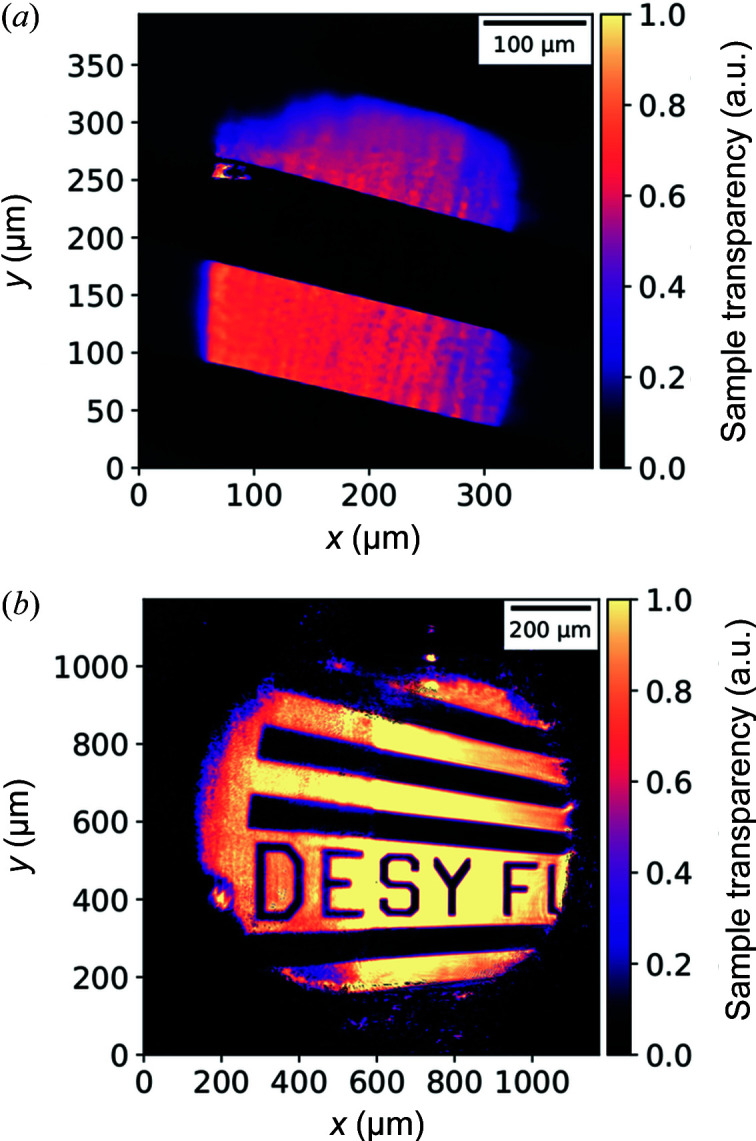
The sample reconstruction transparency of the sample from (*a*) the data measured with the PERCIVAL detector and (*b*) during the experiment described by Kharitonov *et al.* (2021 ▸).

**Figure 4:**
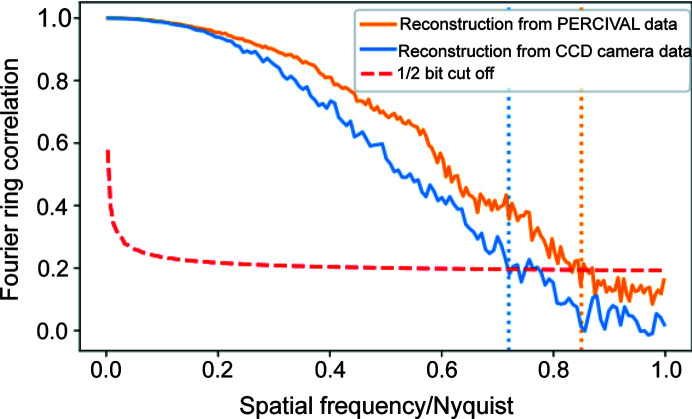
FRC curves calculated from the independent reconstructions shown in Figs. 3 ▸(*a*) in orange and 3(*b*) in blue. The FRC shows a 1.3× improvement of the image obtained from the PERCIVAL detector data, reaching 250 nm.

Typical diffraction patterns from both datasets are shown in Figs. 2 ▸(*c*) and 2 ▸(*d*). It can be seen that the diffraction patterns measured with the PERCIVAL detector registered up to more than twice the diffraction angles and thus contain more high-frequency information. Both measured datasets were processed by an AD-based ptychographical algorithm developed in-house (Kharitonov *et al.*, 2021 ▸) leading to the reconstructions shown in Figs. 3 ▸(*a*) and 3 ▸(*b*). The details of the AD concept and retrieval algorithm can be found elsewhere (Kandel *et al.*, 2019 ▸; Kharitonov *et al.*, 2021 ▸). As a standard estimate for the retrieval resolution, the Fourier ring correlation (FRC) was calculated using independent reconstructions from each dataset shown in Figs. 3 ▸(*a*) and 3 ▸(*b*). FRC estimates the degree of correlation between the frequency spectra of the two independently reconstructed images and allows us to determine the effective resolution of the reconstructions (Banterle *et al.*, 2013 ▸). The FRC curves, shown in Fig. 4 ▸, demonstrate a considerable improvement of the resolution achieved by utilizing the PERCIVAL detector.

Performing ptychography imaging is a twofold challenge demanding high-speed data acquisition in combination with complex analysis routines required for reconstructions. Soft X-rays down to λ = 1.5 nm, available after the upgrade of FLASH (Allaria *et al.*, 2021 ▸), coupled with high-dynamic-range, high-frame-rate soft X-ray detectors such as PERCIVAL and combined with adaptive automatic differentiation (AAD) ptychography (Kharitonov *et al.*, 2021 ▸) open new avenues to study complex engineered materials at FLASH.

## 4. X-ray photon correlation spectroscopy

XPCS is a coherent X-ray scattering technique that probes the dynamics of matter (Lehmkühler *et al.*, 2021 ▸). When a coherent X-ray beam is scattered by a sample, a grainy diffraction pattern, the so-called speckle pattern, is obtained. If the exposed sample volume changes its structure over time, so does the speckle pattern. This is tracked by temporal correlations in a series of speckle patterns in XPCS experiments. In general, the correlations are given by 

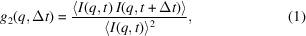

comparing intensities *I* at two different times *t* and *t* + Δ*t*. The averaging is typically performed over detector pixels with equivalent *q* values as well as all times *t*, providing access to average dynamics during exposure.

The correlation function *g*_2_ is connected to the intermediate scattering function *f* that describes the whole temporal evolution of the sample by 

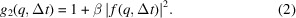

Here, β is the speckle contrast that is 1 for a fully coherent source (*e.g.* optical lasers) and 0 for incoherent radiation. Thus, at least a partially coherent X-ray beam (β > 0) is needed for performing XPCS.

Aside from the coherence properties of the X-ray radiation, the key component in XPCS experiments is the detector. The repetition rate of the detector defines the accessible timescale in XPCS experiments. For hard X-rays, Eiger and LAMBDA detectors have recently become standard for XPCS experiments, allowing (sub-)microsecond timescales to be reached. Faster times have been achieved using prototypical detectors at SRs (Zhang *et al.*, 2018 ▸; Jo *et al.*, 2021 ▸) and using the megahertz repetition rate at the European XFEL (Lehmkühler *et al.*, 2020 ▸; Dallari *et al.*, 2021 ▸). Thanks to its signal-to-noise ratio SNR ∝ *I*_c_(τ_c_)^1/2^ (Falus *et al.*, 2006 ▸) with coherent flux *I*_c_ that is proportional to the brilliance of the light source and the shortest achievable correlation time τ_c_, an increase of brilliance by a factor of *n* will result in access to *n* ^2^ shorter correlation times. Thus, XPCS will benefit significantly from the next generation of SRs, enabling XPCS experiments down to the nanosecond regime.

While hard X-ray XPCS is an established method at modern SRs in particular, with dedicated beamlines at many SRs, only a limited number of soft XPCS studies have been reported. In contrast with hard X-rays, where structural relaxations in liquids and amorphous solids are typically studied, soft XPCS allows the investigation of the dynamics of electronic structures via resonant scattering. Recent examples cover jamming of magnetic domains (Chen *et al.*, 2013 ▸), stability of charge density waves in cuprates (Chen *et al.*, 2016 ▸), slow dynamics in artificial spin ice (Morley *et al.*, 2017 ▸) and spin–stripe dynamics in nickelates close to the onset temperature of superconductivity (Ricci *et al.*, 2021 ▸). Here, the focus was set on very slow relaxation times of several hundred to several thousand seconds. Using a multi-hertz frame rate FastCCD, dynamics of magnetic domains with 0.1 s resolution have recently been reported (Chen *et al.*, 2019 ▸). In contrast, nanosecond timescales have been reached through investigating fast skyrmion dynamics at 1.2 keV at FELs (Seaberg *et al.*, 2017 ▸, 2021 ▸) using a double-pulse visibility spectroscopy approach (Roseker *et al.*, 2018 ▸).

The coherent flux is typically higher for soft X-rays and the speckle size is larger because of the wavelength dependence. Thus, the requirements for XPCS experiments at SRs are even better fulfilled for soft X-rays than for hard X-rays. However, typical soft X-ray area detectors operate with repetition rates in the hertz regime, limiting studies to slow dynamics only, as illustrated also by the examples mentioned previously. Here, PERCIVAL will open the way to sub-second dynamics studies, extending the time range of soft-XPCS by more than two orders of magnitude. Thanks to its pixel size of 27 µm, the conditions for detection of speckles are relaxed, *e.g.* with 50 cm sample-to-detector distance and *E*_ph_ ≃ 800 eV, a beam size of only approximately 30 µm is needed for best performance.

**Figure 5:**
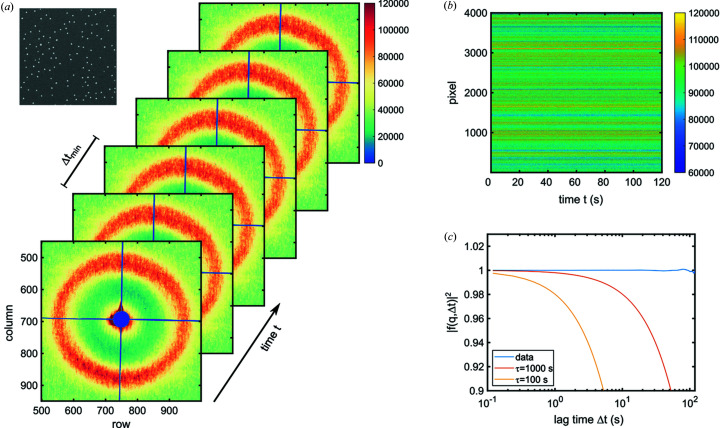
(*a*) Series of speckle patterns measured from a static sample at P04 (PETRA III). The speckle forming the structure factor peak exhibits a single-frame speckle contrast of about 30%. The inset shows a microscope image of the static sample. (*b*) Waterfall plot of intensity over 1000 speckle patterns with a 0.12 s single frame exposure. The data were taken from the structure factor ring in (*a*). (*c*) Intermediate scattering function from the data shown in (*b*). For comparison, correlation functions with relaxation times τ = 1000 s and τ = 100 s are shown, highlighting the high stability during the experiment.

First XPCS datasets taken with PERCIVAL at beamline P04 of PETRA III are shown in Fig. 5 ▸. Fig. 5 ▸(*a*) shows a series of speckle patterns from a static sample. The sample was a spatially disordered nanodot array made from a homogeneous metallic multilayer (Co_1.65 nm_/Pt_2 nm_)_2_ fabricated by nanosphere lithography (Bagschik *et al.*, 2020 ▸). In the experiment, a series of 1000 patterns was taken with an exposure time of Δ*t*_min_ = 0.12 s per pattern. Fig. 5 ▸(*b*) shows a so-called waterfall plot where the intensity in 4000 pixels is plotted as a function of time. As the sample is static, a constant intensity is expected. This is further highlighted in Fig. 5 ▸(*c*) where the intermediate scattering function of the data is given by the blue line. As expected for a static sample, no deviation from unity can be observed, indicating a high stability and good detector performance during the measurement of the XPCS series.

**Figure 6:**
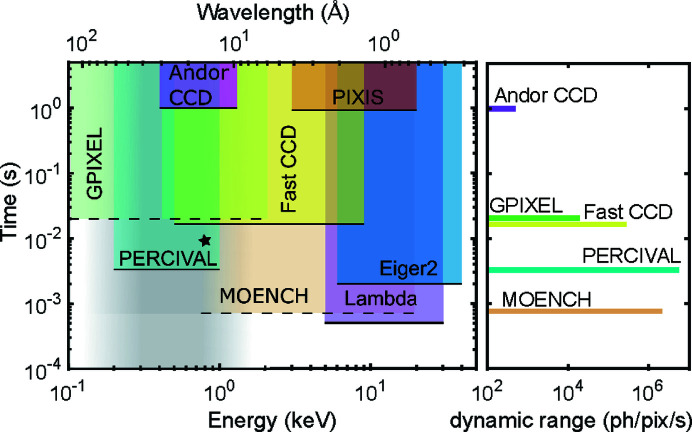
Timescales versus photon energy and/or photon wavelength in the soft and hard X-ray domains. The accessible range for common XPCS detectors are marked. The combination of the PERCIVAL sensor postprocessing (and thus its high sensitivity of low energy photons), together with its high frame rate (up to 300 Hz), will allow the scientific community to access a virtually unknown timescale. The star marks a typical timescale of skyrmion diffusion (size 1 µm) at room temperature (Zázvorka *et al.*, 2019 ▸). The dynamic range is also compared for the detector systems analyzed at the photon energy *E*_ph_ = 700 eV.

With this approach, dynamics of new material classes will become accessible. One example is the thermal diffusion of magnetic skyrmions. As these skyrmions are stable at room temperature, they are candidates for new storage devices (Fert *et al.*, 2013 ▸). Skyrmions may also form dense two-dimensional structures, such as glasses and skyrmion lattices (Huang *et al.*, 2020 ▸). However, little is known about their dynamics and the influence of underlying pinning potentials, skyrmion–skyrmion interactions and external fields. Furthermore, as two-dimensional systems in thin film structures, skyrmion systems may be used as a model system for the kinetics and dynamics of phase transitions in reduced geometries. In particular, whether and how skyrmion lattices and glasses melt is of interest and can be answered by real-time soft XPCS. Recently, diffusion coefficients of skyrmions have been reported using single-particle tracking via magneto-optical Kerr effect (MOKE) microscopy (Zázvorka *et al.*, 2019 ▸). At room temperature, diffusion coefficients were found to be in the range of 10^−12^ m^2^ s^−1^, which corresponds to relaxation times between 10^−2^ s and 1 s for the relevant *q* range. A comparison between different detectors used in soft and hard X-ray experiments is shown in Fig. 6 ▸. Therein, the accessible timescales of the detectors are shown versus the photon energy for which they can be used. The Andor detector is frequently used for soft XPCS at BNL (Chesnel *et al.*, 2008 ▸), the FastCCD covers a broader energy range so that it can also be applied around 8 keV (Falus *et al.*, 2004 ▸). The star marked in Fig. 6 ▸ represents the typical timescale of skyrmion diffusion (size 1 µm) at room temperature (Zázvorka *et al.*, 2019 ▸). As these timescales are inaccessible with standard detectors, the PERCIVAL detector has the potential to become a game changer for studying real-time dynamics with soft X-rays and makes full use of the increased brilliance of next-generation X-ray sources.

## 5. Conclusions and outlook

As mentioned in Section 1, the PERCIVAL system is in its final development phase. Its capabilities at the time of the experiments in summer/fall 2020 have already been described elsewhere (Marras *et al.*, 2021 ▸). The larger dynamic range of the PERCIVAL sensor, and its large active area compared with other detectors, permit the user to register much higher *q* values, carrying more high-frequency information, which is key to achieve higher spatial resolutions. The status of the key parameters – at the time of these experiments – of the system compared with its expected performance is shown in Table 1 ▸.

In the ptychography experiments of Section 3, for instance, a 30% improvement was measured. Also holographic imaging, as discussed in Section 2, benefits from a higher dynamic range since it allows the recording of high-angle scattered photons without overexposing the detector at low angles, achieving a higher resolution. Moreover, in the case of synchrotron radiation applications, the higher frame rate of PERCIVAL allows for higher data throughput than commercially available cameras.

Other fields could also benefit from the features of the PERCIVAL detector system. The soft X-ray XPCS, for instance, has until now been limited to the study of slow dynamics due to the few-hertz frame rate of available cameras. The higher frame rate of PERCIVAL would be able to extend the time range of dynamic studies, as shown in Fig. 6 ▸. Also, the 27 µm pixel size would help to relax some experimental contraints. As discussed in Section 4, the thermal diffusion of magnetic skyrmions is a clear example of a scientific case that could benefit from the use of the PERCIVAL system.

The upcoming redesign of the PERCIVAL chip will overcome the issues already reported by Marras *et al.* (2021 ▸); in particular it should enable both automatic dynamic gain operation in a single image over the full gain range and operation at 300 Hz frame rate and (in ROI mode) beyond. This new design will include the lessons learnt in the experiments listed, together with other characterization campaigns previously reported. Meanwhile, and until the new chip arrives, further experiments using the current version are already scheduled, and improvements in operational performance of the existing sensors continue to become available. All in all, the results shown here demonstrate that PERCIVAL, although still under development, is already capable of providing users with a detector system that significantly improves the performance of available instruments and could enable important break-throughs in the soft X-ray energy range.

## Figures and Tables

**Table 1:** PERCIVAL current status versus aimed goals

Specifications	Gain mode	Current version	Aimed goal
Maximum frame rate (Hz)	–	83.3	300
Noise (e^−^)	Fix – very high	24.39 ± 10.69	–
	Fix – high	64.69 ± 29.53	–
	Fix – med	425.38 ± 231.99	–
	Fix – low	3859 ± 2171	–
	Dynamic	24.39 ± 10.69	15
Full well (e^−^)	Fix – very high	5.75k ± 585	–
	Fix – high	30.5k ± 2k	–
	Fix – med	381k ± 17.6k	–
	Fix – low	3.56M ± 169k	–
	Dynamic	3.1M ± 201k	3.5M

## References

[bb32] Allaria, E., Baboi, N., Baev, K., Beye, M., Brenner, G., Christie, F., Gerth, C., Hartl, I., Honkavaara, K., Manschwetus, B., Mueller-Dieckmann, J., Pan, R., Plönjes-Palm, E., Rasmussen, O., Rönsch-Schulenburg, J., Schaper, L., Schneidmiller, E., Schreiber, S., Tiedtke, K. I., Tischer, M., Toleikis, S., Treusch, R., Vogt, M., Winkelmann, L., Yurkov, M. V. & Zemella, J. (2021). *Proceedings of the 12th International Particle Accelerator Conference (IPAC’21)*, 24–28 May 2021, Campinas, SP, Brazil, pp. 1581–1584. TUPAB086.

[bb47] Bagschik, K., Wagner, J., Buß, R., Riepp, M., Philippi-Kobs, A., Müller, L., Buck, J., Trinter, F., Scholz, F., Seltmann, J., Hoesch, M., Viefhaus, J., Grübel, G., Oepen, H. P. & Frömter, R. (2020). *Opt. Express*, **28**, 7282. 10.1364/OE.382608 32225960

[bb31] Banterle, N., Bui, K. H., Lemke, E. A. & Beck, M. (2013). *J. Struct. Biol.* **183**, 363–367. 10.1016/j.jsb.2013.05.004 23684965

[bb11] Büttner, F., Lemesh, I., Schneider, M., Pfau, B., Günther, C. M., Hessing, P., Geilhufe, J., Caretta, L., Engel, D., Krüger, B., Viefhaus, J., Eisebitt, S. & Beach, G. S. D. (2017). *Nat. Nanotechnol.* **12**, 1040–1044. 10.1038/nnano.2017.178 28967891

[bb10] Caretta, L., Mann, M., Büttner, F., Ueda, K., Pfau, B., Günther, C. M., Hessing, P., Churikova, A., Klose, C., Schneider, M., Engel, D., Marcus, C., Bono, D., Bagschik, K., Eisebitt, S. & Beach, G. S. D. (2018). *Nat. Nanotechnol.* **13**, 1154–1160. 10.1038/s41565-018-0255-3 30224795

[bb18] Chapman, H. N., Barty, A., Marchesini, S., Noy, A., Hau-Riege, S. P., Cui, C., Howells, M. R., Rosen, R., He, H., Spence, J. C. H., Weierstall, U., Beetz, T., Jacobsen, C. & Shapiro, D. (2006). *J. Opt. Soc. Am. A*, **23**, 1179. 10.1364/josaa.23.001179 16642197

[bb39] Chen, S., Guo, H., Seu, K. A., Dumesnil, K., Roy, S. & Sinha, S. K. (2013). *Phys. Rev. Lett.* **110**, 217201. 10.1103/PhysRevLett.110.217201 23745918

[bb43] Chen, X., Farmer, B., Woods, J., Dhuey, S., Hu, W., Mazzoli, C., Wilkins, S., Chopdekar, R., Scholl, A., Robinson, I., De Long, L., Roy, S. & Hastings, J. (2019). *Phys. Rev. Lett.* **123**, 197202. 10.1103/PhysRevLett.123.197202 31765174

[bb40] Chen, X., Thampy, V., Mazzoli, C., Barbour, A., Miao, H., Gu, G., Cao, Y., Tranquada, J., Dean, M. & Wilkins, S. (2016). *Phys. Rev. Lett.* **117**, 167001. 10.1103/PhysRevLett.117.167001 27792368

[bb51] Chesnel, K., Turner, J. J., Pfeifer, M. & Kevan, S. D. (2008). *Appl. Phys. A*, **92**, 431–437.

[bb2] Correa, J., Bayer, M., Göttlicher, P., Lange, S., Marras, A., Niemann, M., Reza, S., Shevyakov, I., Smoljanin, S., Tennert, M., Xia, Q., Viti, M., Wunderer, C. B., Zimmer, M., Dipayan, D., Guerrini, N., Marsh, B., Sedgwick, I., Turchetta, R., Cautero, G., Giuressi, D., Khromova, A., Pinaroli, G., Menk, R., Stebel, L., Fan, R., Marchal, J., Pedersen, U., Rees, N., Steadman, P., Sussmuth, M., Tartoni, N., Yousef, H., Hyun, H. J., Kim, K., Rah, S. & Graafsma, H. (2016*a*). *J. Instrum.* **11**, C02090.

[bb3] Correa, J., Marras, A., Wunderer, C. B., Göttlicher, P., Lange, S., Reza, S., Shevyakov, I., Tennert, M., Niemann, M., Hirsemann, H., Smoljanin, S., Supra, J., Xia, Q., Zimmer, M., Allahgholi, A., Gloskovskii, A., Viefhaus, J., Scholz, F., Seltmann, J., Klumpp, S., Cautero, G., Giuressi, D., Khromova, A., Menk, R., Pinaroli, G., Stebel, L., Rinaldi, S., Zema, N., Catone, D., Pedersen, U., Tartoni, N., Guerrini, N., Marsh, B., Sedgwick, I., Nicholls, T., Turchetta, R., Hyun, H. J., Kim, K. S., Rah, S. Y., Hoenk, M. E., Jewell, A. D., Jones, T. J., Nikzad, S. & Graafsma, H. (2016*b*). *J. Instrum.* **11**, C12032.

[bb37] Dallari, F., Jain, A., Sikorski, M., Möller, J., Bean, R., Boesenberg, U., Frenzel, L., Goy, C., Hallmann, J., Kim, Y., Lokteva, I., Markmann, V., Mills, G., Rodriguez-Fernandez, A., Roseker, W., Scholz, M., Shayduk, R., Vagovic, P., Walther, M., Westermeier, F., Madsen, A., Mancuso, A. P., Grübel, G. & Lehmkühler, F. (2021). *IUCrJ*, **8**, 775–783. 10.1107/S2052252521006333 PMC8420773 34584738

[bb7] Eisebitt, S., Lörgen, M., Eberhardt, W., Lüning, J., Andrews, S. & Stöhr, J. (2004). *Appl. Phys. Lett.* **84**, 3373–3375.

[bb30] Faatz, B., Plönjes, E., Ackermann, S., Agababyan, A., Asgekar, V., Ayvazyan, V., Baark, S., Baboi, N., Balandin, V., Bargen, N., Bican, Y., Bilani, O., Bödewadt, J., Böhnert, M., Böspflug, R., Bonfigt, S., Bolz, H., Borges, F., Borkenhagen, O., Brachmanski, M., Braune, M., Brinkmann, A., Brovko, O., Bruns, T., Castro, P., Chen, J., Czwalinna, M. K., Damker, H., Decking, W., Degenhardt, M., Delfs, A., Delfs, T., Deng, H., Dressel, M., Duhme, H., Düsterer, S., Eckoldt, H., Eislage, A., Felber, M., Feldhaus, J., Gessler, P., Gibau, M., Golubeva, N., Golz, T., Gonschior, J., Grebentsov, A., Grecki, M., Grün, C., Grunewald, S., Hacker, K., Hänisch, L., Hage, A., Hans, T., Hass, E., Hauberg, A., Hensler, O., Hesse, M., Heuck, K., Hidvegi, A., Holz, M., Honkavaara, K., Höppner, H., Ignatenko, A., Jäger, J., Jastrow, U., Kammering, R., Karstensen, S., Kaukher, A., Kay, H., Keil, B., Klose, K., Kocharyan, V., Köpke, M., Körfer, M., Kook, W., Krause, B., Krebs, O., Kreis, S., Krivan, F., Kuhlmann, J., Kuhlmann, M., Kube, G., Laarmann, T., Lechner, C., Lederer, S., Leuschner, A., Liebertz, D., Liebing, J., Liedtke, A., Lilje, L., Limberg, T., Lipka, D., Liu, B., Lorbeer, B., Ludwig, K., Mahn, H., Marinkovic, G., Martens, C., Marutzky, F., Maslocv, M., Meissner, D., Mildner, N., Miltchev, V., Molnar, S., Mross, D., Müller, F., Neumann, R., Neumann, P., Nölle, D., Obier, F., Pelzer, M., Peters, H., Petersen, K., Petrosyan, A., Petrosyan, G., Petrosyan, L., Petrosyan, V., Petrov, A., Pfeiffer, S., Piotrowski, A., Pisarov, Z., Plath, T., Pototzki, P., Prandolini, M. J., Prenting, J., Priebe, G., Racky, B., Ramm, T., Rehlich, K., Riedel, R., Roggli, M., Röhling, M., Rönsch-Schulenburg, J., Rossbach, J., Rybnikov, V., Schäfer, J., Schaffran, J., Schlarb, H., Schlesselmann, G., Schlösser, M., Schmid, P., Schmidt, C., Schmidt-Föhre, F., Schmitz, M., Schneidmiller, E., Schöps, A., Scholz, M., Schreiber, S., Schütt, K., Schütz, U., Schulte-Schrepping, H., Schulz, M., Shabunov, A., Smirnov, P., Sombrowski, E., Sorokin, A., Sparr, B., Spengler, J., Staack, M., Stadler, M., Stechmann, C., Steffen, B., Stojanovic, N., Sychev, V., Syresin, E., Tanikawa, T., Tavella, F., Tesch, N., Tiedtke, K., Tischer, M., Treusch, R., Tripathi, S., Vagin, P., Vetrov, P., Vilcins, S., Vogt, M., Wagner, A. Z., Wamsat, T., Weddig, H., Weichert, G., Weigelt, H., Wentowski, N., Wiebers, C., Wilksen, T., Willner, A., Wittenburg, K., Wohlenberg, T., Wortmann, J., Wurth, W., Yurkov, M., Zagorodnov, I. & Zemella, J. (2016). *New J. Phys.* **18**, 062002.

[bb52] Falus, P., Borthwick, M. A. & Mochrie, S. G. J. (2004). *Rev. Sci. Instrum.* **75**, 4383–4400.

[bb38] Falus, P., Lurio, L. B. & Mochrie, S. G. J. (2006). *J. Synchrotron Rad.* **13**, 253–259. 10.1107/S0909049506006789 16645251

[bb48] Fert, A., Cros, V. & Sampaio, J. (2013). *Nat. Nanotechnol.* **8**, 152–156. 10.1038/nnano.2013.29 23459548

[bb24] Gao, Z., Holler, M., Odstrcil, M., Menzel, A., Guizar-Sicairos, M. & Ihli, J. (2020). *Chem. Commun.* **56**, 13373–13376. 10.1039/d0cc06101h 33030473

[bb12] Gerlinger, K., Pfau, B., Büttner, F., Schneider, M., Kern, L., Fuchs, J., Engel, D., Günther, C. M., Huang, M., Lemesh, I., Caretta, L., Churikova, A., Hessing, P., Klose, C., StrüBer, C., Schmising, C. K., Huang, S., Wittmann, A., Litzius, K., Metternich, D., Battistelli, R., Bagschik, K., Sadovnikov, A., Beach, G. S. D. & Eisebitt, S. (2021). *Appl. Phys. Lett.* **118**, 192403.

[bb49] Huang, P., Schönenberger, T., Cantoni, M., Heinen, L., Magrez, A., Rosch, A., Carbone, F. & Rønnow, H. M. (2020). *Nat. Nanotechnol.* **15**, 892. 10.1038/s41565-020-00774-3 32901151

[bb35] Jo, W., Westermeier, F., Rysov, R., Leupold, O., Schulz, F., Tober, S., Markmann, V., Sprung, M., Ricci, A., Laurus, T., Aschkan, A., Klyuev, A., Trunk, U., Graafsma, H., Grübel, G. & Roseker, W. (2021). *IUCrJ*, **8**, 124–130. 10.1107/S2052252520015778 PMC7792991 33520248

[bb25] Kandel, S., Maddali, S., Allain, M., Hruszkewycz, S. O., Jacobsen, C. & Nashed, Y. S. G. (2019). *Opt. Express*, **27**, 18653. 10.1364/OE.27.018653 PMC6825598 31252805

[bb16] Kfir, O., Zayko, S., Nolte, C., Sivis, M., Möller, M., Hebler, B., Arekapudi, S. S. P. K., Steil, D., Schäfer, S., Albrecht, M., Cohen, O., Mathias, S. & Ropers, C. (2017). *Sci. Adv.* **3**, eaao4641. 10.1126/sciadv.aao4641 PMC5732000 29250601

[bb26] Kharitonov, K., Mehrjoo, M., Ruiz-Lopez, M., Keitel, B., Kreis, S., Seyrich, M., Pop, M. & Plönjes, E. (2021). *Opt. Express*, **29**, 22345. 10.1364/OE.426931 34266001

[bb36] Lehmkühler, F., Dallari, F., Jain, A., Sikorski, M., Möller, J., Frenzel, L., Lokteva, I., Mills, G., Walther, M., Sinn, H., Schulz, F., Dartsch, M., Markmann, V., Bean, R., Kim, Y., Vagovic, P., Madsen, A., Mancuso, A. P. & Grübel, G. (2020). *Proc. Natl. Acad. Sci. USA.* **117**, 24110–24116. 10.1073/pnas.2003337117 PMC7533660 32934145

[bb33] Lehmkühler, F., Roseker, W. & Grübel, G. (2021). *Appl. Sci.* **11**, 6179.

[bb17] Marchesini, S. (2007). *Rev. Sci. Instrum.* **78**, 049901.

[bb4] Marras, A., Correa, J., Lange, S., Vardanyan, V., Gerhardt, T., Kuhn, M., Krivan, F., Shevyakov, I., Zimmer, M., Hoesch, M., Bagschik, K., Scholz, F., Guerrini, N., Marsh, B., Sedgwick, I., Cautero, G., Giuressi, D., Iztok, G., Menk, R. H., Scarcia, M., Stebel, L., Nicholls, T., Nichols, W., Pedersen, U. K., Shikhaliev, P., Tartoni, N., Hyun, H., Kim, S., Kim, K., Rah, S., Dawiec, A., Orsini, F., Pinaroli, G., Greer, A., Aplin, S., Jewell, A. D., Jones, T. J., Nikzad, S., Hoenk, M. E., Okrent, F., Graafsma, H. & Wunderer, C. B. (2021). *J. Synchrotron Rad.* **28**, 131–145.

[bb6] McNulty, I., Kirz, J., Jacobsen, C., Anderson, E. H., Howells, M. R. & Kern, D. P. (1992). *Science*, **256**, 1009–1012. 10.1126/science.256.5059.1009 17795006

[bb41] Morley, S. A., Alba Venero, D., Porro, J. M., Riley, S. T., Stein, A., Steadman, P., Stamps, R. L., Langridge, S. & Marrows, C. H. (2017). *Phys. Rev. B*, **95**, 104422.

[bb15] Pfau, B. & Eisebitt, S. (2016). *Synchrotron Light Sources and Free-Electron Lasers*, pp. 1093–1133. Springer.

[bb20] Pfeiffer, F. (2018). *Nat. Photon.* **12**, 9–17.

[bb28] Plönjes, E., Faatz, B., Kuhlmann, M. & Treusch, R. (2016). *AIP Conf. Proc.* **1741**, 020008.

[bb42] Ricci, A., Poccia, N., Campi, G., Mishra, S., Müller, L., Joseph, B., Shi, B., Zozulya, A., Buchholz, M., Trabant, C., Lee, J. C., Viefhaus, J., Goedkoop, J. B., Nugroho, A. A., Braden, M., Roy, S., Sprung, M. & Schüßler-Langeheine, C. (2021). *Phys. Rev. Lett.* **127**, 057001. 10.1103/PhysRevLett.127.057001 34397237

[bb27] Rose, M., Dzhigaev, D., Senkbeil, T., von Gundlach, A. R., Stuhr, S., Rumancev, C., Besedin, I., Skopintsev, P., Viefhaus, J., Rosenhahn, A. & Vartanyants, I. A. (2017). *J. Phys. Conf. Ser.* **849**, 012027.

[bb46] Roseker, W., Hruszkewycz, S. O., Lehmkühler, F., Walther, M., Schulte-Schrepping, H., Lee, S., Osaka, T., Strüder, L., Hartmann, R., Sikorski, M., Song, S., Robert, A., Fuoss, P. H., Sutton, M., Stephenson, G. B. & Grübel, G. (2018). *Nat. Commun.* **9**, 1704. 10.1038/s41467-018-04178-9 PMC5923200 29703980

[bb29] Ruiz-Lopez, M. (2019). *Proc. SPIE*, **11038**, 110380F.

[bb19] Saxton, W. O. & Baumeister, W. (1982). *J. Microsc.* **127**, 127–138. 10.1111/j.1365-2818.1982.tb00405.x 7120365

[bb44] Seaberg, M., Holladay, B., Lee, J., Sikorski, M., Reid, A., Montoya, S., Dakovski, G., Koralek, J., Coslovich, G., Moeller, S., Schlotter, W., Streubel, R., Kevan, S., Fischer, P., Fullerton, E., Turner, J., Decker, F., Sinha, S., Roy, S. & Turner, J. (2017). *Phys. Rev. Lett.* **119**, 067403. 10.1103/PhysRevLett.119.067403 28949638

[bb45] Seaberg, M. H., Holladay, B., Montoya, S. A., Zheng, X. Y., Lee, J. C. T., Reid, A. H., Koralek, J. D., Shen, L., Esposito, V., Coslovich, G., Walter, P., Zohar, S., Thampy, V., Lin, M. F., Hart, P., Nakahara, K., Streubel, R., Kevan, S. D., Fischer, P., Colocho, W., Lutman, A., Decker, F., Fullerton, E. E., Dunne, M., Roy, S., Sinha, S. K. & Turner, J. J. (2021). *Phys. Rev. Res.* **3**, 033249.

[bb23] Shi, X., Fischer, P., Neu, V., Elefant, D., Lee, J. C. T., Shapiro, D. A., Farmand, M., Tyliszczak, T., Shiu, H., Marchesini, S., Roy, S. & Kevan, S. D. (2016). *Appl. Phys. Lett.* **108**, 094103.

[bb8] Stöhr, J. & Siegmann, H. C. (2006). *Magnetism: From Fundamentals to Nanoscale Dynamics*, Vol. 152 of *Springer Series in Solid-State Sciences*. Springer.

[bb22] Sun, T., Sun, G., Yu, F., Mao, Y., Tai, R., Zhang, X., Shao, G., Wang, Z., Wang, J. & Zhou, J. (2021). *ACS Nano*, **15**, 1475–1485. 10.1021/acsnano.0c08891 33356135

[bb21] Thibault, P., Dierolf, M., Menzel, A., Bunk, O., David, C. & Pfeiffer, F. (2008). *Science*, **321**, 379–382. 10.1126/science.1158573 18635796

[bb9] Turnbull, L. A., Birch, M. T., Laurenson, A., Bukin, N., Burgos-Parra, E. O., Popescu, H., Wilson, M. N., Stefančič, A., Balakrishnan, G., Ogrin, F. Y. & Hatton, P. D. (2021). *ACS Nano*, **15**, 387–395. 10.1021/acsnano.0c07392 33119252

[bb14] Viefhaus, J., Scholz, F., Deinert, S., Glaser, L., Ilchen, M., Seltmann, J., Walter, P. & Siewert, F. (2013). *Nucl. Instrum. Methods Phys. Res. A*, **710**, 151–154.

[bb5] Wunderer, C. B., Correa, J., Marras, A., Aplin, S., Boitrelle, B., Goettlicher, P., Krivan, F., Kuhn, M., Lange, S., Niemann, M., Okrent, F., Shevyakov, I., Zimmer, M., Guerrini, N., Marsh, B., Sedgwick, I., Cautero, G., Giuressi, D., Gregori, I., Pinaroli, G., Menk, R., Stebel, L., Greer, A., Nicholls, T., Pedersen, U. K., Tartoni, N., Hyun, H., Kim, K., Rah, S. & Graafsma, H. (2019). *J. Instrum.* **14**, C01006.

[bb1] Wunderer, C. B., Marras, A., Bayer, M., Glaser, L., Göttlicher, P., Lange, S., Pithan, F., Scholz, F., Seltmann, J., Shevyakov, I., Smoljanin, S., Viefhaus, J., Viti, M., Xia, Q., Zimmer, M., Klumpp, S., Gasiorek, P., Guerrini, N., Marsh, B., Sedgwick, I., Turchetta, R., Cautero, G., Farina, S., Giuressi, D., Menk, R., Stebel, L., Yousef, H., Marchal, J., Nicholls, T., Tartoni, N. & Graafsma, H. (2014). *J. Instrum.* **9**, C03056.

[bb50] Zázvorka, J., Jakobs, F., Heinze, D., Keil, N., Kromin, S., Jaiswal, S., Litzius, K., Jakob, G., Virnau, P., Pinna, D., Everschor-Sitte, K., Rózsa, L., Donges, A., Nowak, U. & Kläui, M. (2019). *Nat. Nanotechnol.* **14**, 658–661. 10.1038/s41565-019-0436-8 31011220

[bb34] Zhang, Q., Dufresne, E. M., Narayanan, S., Maj, P., Koziol, A., Szczygiel, R., Grybos, P., Sutton, M. & Sandy, A. R. (2018). *J. Synchrotron Rad.* **25**, 1408–1416. 10.1107/S1600577518009074 30179180

